# Granulomatous Dermatitis and Systemic Disease: An Association to Consider

**DOI:** 10.1155/2020/3281380

**Published:** 2020-09-30

**Authors:** Alberto Corrà, Lavinia Quintarelli, Alice Verdelli, Francesca Portelli, Daniela Massi, Marzia Caproni

**Affiliations:** ^1^Dermatology Unit, Department of Health Sciences, University of Florence, Florence, Italy; ^2^Department of Experimental and Clinical Biomedical Sciences “Mario Serio”, University of Florence, Viale Morgagni 50, 50134 Florence, Italy; ^3^Division of Pathological Anatomy, Department of Surgical and Translational Medicine, University of Florence, Florence, Italy

## Abstract

Granuloma annulare (GA) and interstitial granulomatous dermatitis (IGD) are granulomatous dermatoses with variable clinical appearances. GA is associated with diabetes mellitus, metabolic syndrome, chronic infections, and malignancies, while two Japanese reports described unusual cases of interstitial-type GA in setting of Sjogren syndrome. IGD was associated with rheumatoid arthritis, systemic lupus erythematosus, and autoantibodies. We report a case series of six patients with GA or IGD. Half of the patients were diagnosed with Sjogren syndrome, while all of them presented ANA positivity and the majority reported arthralgia. In many cases, GA showed interstitial-type histology, arising challenges in differential diagnosis with IGD. The overlap of clinical and histological features of GA and IGD can be explained considering them as a broad disease spectrum, including also the other forms of reactive granulomatous dermatitis. These conditions should be considered as an indicator of possible systemic disorders or other immunological dyscrasias, for which patients must be screened. Sjogren syndrome may be associated to GA also in Caucasians.

## 1. Introduction

Granulomatous dermatoses are a group of clinically and histologically heterogeneous inflammatory skin conditions including granuloma annulare (GA) and reactive granulomatous dermatitis (RGD).

GA was described in association with diabetes mellitus, chronic infections, malignancies, and thyroid autoimmune diseases [1], while in the last decade association with autoimmune systemic diseases was reported in Japanese population. Among these, two GA cases in association with Sjogren syndrome are as follows: one with dermatomyositis and four in the setting of systemic sclerosis or morphoea [2–8]. Clinically, GA erupts usually with ring-like erythematous plaques, scattered coalescing papules, or nodules. While the localized GA classically appears on the hands and feet, the generalized form involves extremities and trunk, and the subcutaneous form usually involves the lower extremities in children. Perforating, patched, macular, pustular, and palmoplantar are rare variants. On histology, mucin with a palisading or interstitial configuration of granulomatous inflammation represents the typical finding [6]. The palisading pattern appears as dermal palisading histiocytes and lymphocytes surrounding a central zone of necrobiotic collagen in upper and middle dermis. In opposition, the interstitial pattern consists of histiocytes scattered around collagen bundles and blood vessels [9].

Under the term RGD, in 2015, Rosenbach et al. unified several granulomatous inflammatory skin reactions with similar clinical and histological features, including palisaded neutrophilic granulomatous dermatitis (PNGD), interstitial granulomatous dermatitis (IGD), and interstitial granulomatous drug reaction (IGDR) [10]. IGD is described in association with autoimmune diseases like arthritis, systemic lupus erythematous, and autoimmune hepatitis, while Verneuil et al. highlighted also the correlation with autoantibodies or rheumatic symptoms like arthralgia. Clinically, it presents with asymptomatic papules and plaques, varying in colour from skin-coloured to erythematous and frequently located in lateral upper trunk, axillae, and proximal upper limbs. Linear, cord-like nodular lesion called “the rope sign” is considered a characteristic manifestation of IGD, despite the low frequency; in fact, it occurs in 9-10% of the cases [11–14]. Histology shows scattered dermal histiocytes among and around foci of degenerated collagen, often rimming altered collagen fibers which may be “floating” (“floating sign”), while usually no vasculitis or mucin are detected.

We present the series of six GA or RGD patients referred to our centre in 2019, describing clinical and histological features, serological findings, and associations.

## 2. Materials and Methods

Six Italian patients were diagnosed with GA or IGD at our institution over the last year; clinical and histological data were collected, as well as serological analysis for autoantibodies and assessment of comorbidities. In addition, PubMed search was performed focusing on the associations of these granulomatous manifestations.

## 3. Results

**Figure 1:**
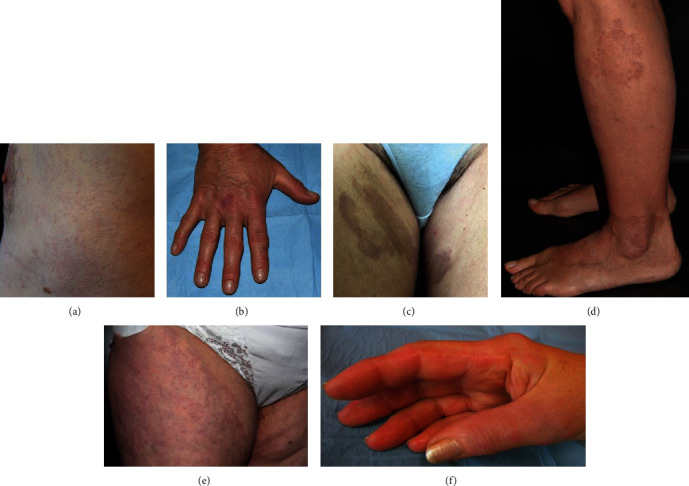
Clinical presentation of skin granulomatous diseases: (a) nonconfluent, skin-coloured papules on lateral trunk; (b, e, f) erythematous papules/nodules on hands and thighs; (c) brownish annular patches on thighs; (d) erythematous papules/nodules with signs of confluence in annular disposition.

**Figure 2:**
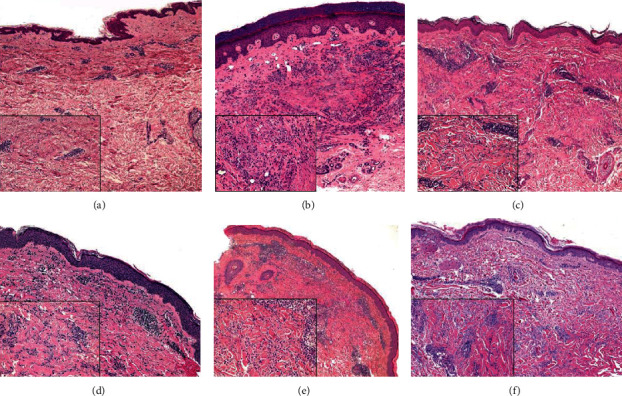
Histological picture in lesional skin samples. (a, f) Interstitial granulomatous dermatitis. Hypercellular “busy” dermis due to increased numbers of inflammatory cells, mainly represented by histiocytes and lymphocytes. They are arranged around vessels and between collagen bundles. A slight increased amount of interstitial mucin is noted in (f) ((a) original magnification ×5 and inset ×10, hematoxylin and eosin; (f) original magnification ×5 and inset ×20, hematoxylin and eosin). (b, e) Necrobiotic (collagenolytic) pattern of granuloma annulare. In the superficial and middermis, an area of necrobiosis is surrounded by a rim of peripheral histiocytes, lymphocytes, and variable numbers of multinucleate giant cells. A perivascular inflammatory infiltrate is also noted ((b) original magnification ×5 and inset ×20, hematoxylin and eosin; (e) original magnification ×5 and inset ×20, hematoxylin and eosin). (c, d) Interstitial pattern of granuloma annulare. Lymphohistiocytic inflammatory dermal infiltration among collagen fibers and mucin deposits ((c) original magnification ×5 and inset X20, hematoxylin and eosin; (d) original magnification ×5 and inset ×20, hematoxylin and eosin).

Clinical and histological data are summarized in Table 1. Mean age at diagnosis was 65, and almost all of them were women (5 of 6). Four patients presented multiple erythematous papules or nodules located mainly on thighs, axillae, and dorsal surface of the hands (Figures 1(b), 1(e), 1(d), and 1(f)). In three of them, some lesions were confluent in annular disposition, clinically compatible with GA, while the fourth patient lacked lesions' confluence (Figure 1(f)); all of them had histological confirmation for GA. One patient presented brownish patches confluent in ring-like form on inner surface of the thighs (Figure 1(c)). Differently, a patient presented skin-coloured papules, with no erythema and no confluence, located at gluteus, hips, axillae, and inner surface of upper limbs (Figure 1(a)). This case was clinically more consistent with IGD. Lesions were symmetric in all cases, while ulceration, excoriation, and cord-like aspect were absent. The patient diagnosed with IGD complained of itch; the other patients did not report skin symptoms. On histologic examination, four cases were consistent with GA, necrobiotic (collagenolytic), or interstitial form. The necrobiotic pattern was characterized by palisading histiocytes and variable number of lymphocytes and neutrophils around a central zone of degenerated collagen fibers and mucin. The interstitial pattern showed collections of histiocytes and lymphocytes dispersed between collagen fibers with interstitial mucin and around blood vessels. In one case, the histological picture of a biopsy taken three years before was consistent with IGD, whereas the second biopsy taken to address the correct diagnosis was compatible with GA (Figure 2(f)). In another case, differentiating interstitial GA from IGD was histologically challenging, and the diagnosis required clinical correlation (Figure 1(a)). Regarding the comorbidities, four of six patients (66%) complained of mild or moderate arthralgia, but none of them was previously investigated for arthritis. Three patients (50%) were diagnosed with Sjogren syndrome, while antinucleus antibodies were positive in all patients with titre ranging from 1 : 80 to 1 : 2560. One patient was already in treatment with hydroxychloroquine (HCQ) for SS and lesions improved after addition of methotrexate, while two cases improved with topical steroids application for 4 weeks. A patient in treatment with HCQ showed complete regression after topical steroids application. Finally, two patients started a treatment with HCQ and topical steroids: one of them had complete disappearing of the lesions, while the other had no improvements after one year.

## 4. Discussion

In our case series, five patients were diagnosed with GA. Of them, two were affected by SS: the concomitance of these conditions can possibly be explained by a common pathogenetic link, instead of representing a simple incidental finding. This hypothesis finds support in literature in two similar cases described in Japanese population [2, 3]. However, this possible correlation needs to be clarified collecting data on larger populations. Exceptionally, a patient was diagnosed with interstitial-type GA three years after receiving IGD diagnosis based on the histologic examination of the first biopsy: this is more probably due to a challenging differential diagnosis than to the appearance of both conditions within three years. Indeed, the concurrence of SS, autoimmune thyroiditis, arthralgia, and ANA positivity in this patient could not suggest a diagnosis, as both GA and IGD were reported in association with those comorbidities [15]. Finally, the patient diagnosed with IGD had history of arthralgia, psoriasis, and polymyalgia rheumatica (PR). To date, no cases of association with PR and psoriasis are described.

According to recent data, our cohort shows that GA shares different features with IGD and, more in general, with all RGDs. Indeed, associations of GA with systemic autoimmune conditions are observed, in addition to the previously described correlations with metabolic, endocrine, and neoplastic diseases. Therefore, the spectrum of disease potentially associated with skin granulomatous manifestations such as GA and IGD is similar. Also, the histologic detection of interstitial pattern of dermal infiltration is a common feature, instead of the classic palisading disposition. Two of five patients diagnosed with GA described in our series presented the interstitial-type histology. Differentiating RGD from interstitial form of GA can be challenging when biopsy shows loosely arranged histiocytes and focal mucin deposits: in this setting, clinical correlation is useful to address the correct diagnosis [16].

GA and RGDs can therefore be considered part of the same disease spectrum, as possible, nonspecific reactive manifestation of systemic diseases or serologic immune abnormalities such as ANA. Different systemic conditions could present similar cutaneous manifestations due to a common pathogenesis, depending possibly on immune complexes deposition leading to granulomatous-type inflammation with collagen damaging [1]. Including GA among the RGD group may be a hypothesis to consider, while the growing number of associations reported emphasizes the importance to screen the patients for underlying systemic disorders.

In addition, the high frequency (50%) of patients diagnosed with Sjogren syndrome suggests a stronger correlation with skin granulomatous diseases not only in Asian people, as previously reported, but also in Caucasian patients. Arthralgia should be carefully investigated when reported, as RGDs are associated with arthritis. Therefore, a management in concert with rheumatologists is needed.

A limitation of our work is the limited number of subjects. However, other studies are needed to confirm the association between granulomatous dermatitis and systemic diseases in order to start the correct multidisciplinary management and a proper treatment.

## Figures and Tables

**Table 1:** Features of patients.

Patient	Age	Sex	Clinical presentation	Histology	Diagnosis	Comorbidities	Serum findings	Treatment/response
1	70	F	Nodular lesions on external surface of thighs Erythematous papules and plaques on back of the hands and fingers (Figure 1(f))	First biopsy (thigh): loosely arranged lymphocytes and histiocytes scattered in superficial and middermis with focal mucin deposits (Figure 2(f)) Second biopsy (hands): necrobiosis area surrounded by a rim of peripheral palisading histiocytes, lymphocytes, and multinucleated giant cells associated with a slight perivascular inflammatory infiltrate in superficial and middermis	First biopsy: IGD Second biopsy: GA	Sjogren syndrome Arthralgia Hypoacusis Type 2 diabetes Hashimoto's thyroiditis Pleuritis	ANA 1 : 2560 SS-A, SS-B	In treatment with hydroxychloroquine 200 mg/die Methotrexate 7.5 mg/week added Improvements with no CR
2	68	M	Nonconfluent, skin-coloured papules of 2-5 mm diameter located on the hips, gluteus, axillae, and inner surface of upper limbs with mild itching (Figure 1(a))	Gluteus skin biopsy: interstitial and perivascular lymphocytes and histiocytes dispersed among the collagen fibers and focal mucin deposits (Figure 2(a))	IGD	Polymyalgia rheumatica Arthralgia Psoriasis Macular degeneration	ANA 1 : 320	Topical steroids with partial response
3	42	F	Erythematous papules confluent in ring-like fashions with central resolution located on the thighs, arms, and axillae (Figure 1(d))	Lymphocytes and histiocytes dispersed among collagen fibers (Figure 2(c))	GA, incomplete/interstitial form	Arthralgia Allergic rhinitis	ANA 1 : 160 Hypergammaglobulinemia	Hydroxychloroquine and topical steroids
4	75	F	Erythematous papules, some with annular disposition, located on the thighs (Figure 1(e))	Moderate lympho-histiocytic inflammatory infiltrate in superficial ad middermis, with perivascular and interstitial distribution among degenerating collagen fibers and mucin deposits (Figure 2(d))	GA, incomplete/interstitial form	Sjogren syndrome Hypothyroidism Osteoporosis Colon diverticulosis Gallstones	ANA 1 : 640 SS-A IgM monoclonal gammopathy	—
5	65	F	Slightly erythematous and confluent nodules localized on the elbows and back of the hands (Figure 1(b))	Hand skin biopsy: lymphocytes, histiocytes, and several multinucleated giant cells in a palisading pattern surrounding areas of degenerated collagen fibers and mucin in the superficial dermis (Figure 2(b))	GA, necrobiotic pattern	Sjogren syndrome	ANA 1 : 80	—
6	70	F	Brownish hardened patches, roundish, and confluent with a slightly hypopigmented central area, located on inner surfaces of the thighs (Figure 1(c))	Thigh skin biopsy: limpho-histiocytic dermal infiltrate surrounding degenerated collagen areas and perivascular (Figure 2(e))	GA, necrobiotic pattern	Arthralgia	ANA 1 : 160 Hypogammaglobulinemia	Hydroxychloroquine plus topical steroids CR in six months Arthralgia resolved

IGD: interstitial granulomatous dermatitis; GA: granuloma annulare; ANA: anti-nucleus antibodies; ENA: antiextractable nuclear antigens autoantibodies; PR: partial response (lesions improved but not disappeared or incomplete disappearing); CR: complete response.

## Data Availability

The clinical images used to support the findings of this study are included. The histologic sample pictures used to support the findings of this study are included within the article. The clinical information on patients' history used to support the findings of this study have not been made available because electronic health record systems are not accessible for obvious reasons of privacy.
